# Social cognition in children and adolescents with epilepsy: A meta-analysis

**DOI:** 10.3389/fpsyt.2022.983565

**Published:** 2022-09-15

**Authors:** Yang Sun, Jing Zhao, PanWen Zhao, Hui Zhang, JianGuo Zhong, PingLei Pan, GenDi Wang, ZhongQuan Yi, LiLi Xie

**Affiliations:** ^1^Department of Clinical Laboratory, The Fourth Affiliated Hospital of Nantong University, The First People's Hospital of Yancheng, Yancheng, Jiangsu, China; ^2^Department of Central Laboratory, The Sixth Affiliated Hospital of Nantong University, Yancheng Third People's Hospital, Yancheng, Jiangsu, China; ^3^Department of Neurology, The Sixth Affiliated Hospital of Nantong University, Yancheng Third People's Hospital, Yancheng, Jiangsu, China; ^4^Department of Neurology, The Sixth Affiliated Hospital of Nantong University, Yancheng Third People's Hospital, Yancheng, Jiangsu, China; ^5^Department of Central Laboratory, The Sixth Affiliated Hospital of Nantong University, Yancheng Third People's Hospital, Yancheng, Jiangsu, China

**Keywords:** children and adolescents, epilepsy, theory of mind, facial emotion recognition, meta-analysis, cognitive, affective

## Abstract

Many studies have investigated impairments in two key domains of social cognition (theory of mind [ToM] and facial emotion recognition [FER]) in children and adolescents with epilepsy. However, inconsistent conclusions were found. Our objective was to characterize social cognition performance of children and adolescents with epilepsy. A literature search was conducted using Web of Science, PubMed, and Embase databases. The article retrieval, screening, quality assessment (Newcastle-Ottawa-Scale), and data extraction were performed independently by two investigators. A random-effects model was used to examine estimates. The meta-analysis included 19 studies, with a combined sample of 623 children and adolescents with epilepsy (mean [SD] age, 12.13 [2.62] years; 46.1% female) and 677 healthy controls [HCs]) (mean [SD] age, 11.48 [2.71] years; 50.7% female). The results revealed that relative to HCs, children and adolescents with epilepsy exhibited deficits in ToM (*g* = −1.08, 95% *CI* [−1.38, −0.78], *p* < 0.001, the number of studies [*k*] = 13), FER (*g* = −0.98, 95% *CI* [−1.33, −0.64], *p* < 0.001, *k* = 12), and ToM subcomponents (cognitive ToM: *g* = −1.04, 95% *CI* [−1.35, −0.72], *p* < 0.001, *k* = 12] and affective ToM: *g* = −0.73, 95% *CI* [−1.12, −0.34], *p* < 0.001, *k* = 8). In addition, there were no statistically significant differences in social cognition deficits between children and adolescents with focal epilepsy and generalized epilepsy. Meta-regressions confirmed the robustness of the results. These quantitative results further deepen our understanding of the two core domains of social cognition in children and adolescents with epilepsy and may assist in the development of cognitive interventions for this patient population.

**Systematic review registration:** https://inplasy.com/inplasy-2022-3-0011/, identifier INPLASY202230011.

## Highlights

- Children and adolescents with epilepsy affect social cognition—theory of mind (ToM) and facial emotion recognition (FER).
- We quantify the magnitude of deficits in ToM and FER in children and adolescents with epilepsy.
- Childhood focal and generalized epilepsy show similar deficits in social cognition.
- This may inform the development of structured cognitive interventions for this patient population.

## Introduction

Epilepsy, characterized by chronic, unprovoked and recurrent seizures (1), is a common neurological condition and usually has its onset in early development (2, 3). An estimated 50 million people worldwide suffer from epileptic seizures (4), with more than half of these cases beginning in childhood and adolescence (5). Recent studies suggests that children and adolescents with epilepsy frequently have psychosocial dysfunction, which commonly lead to pervasive social problems, such as severe economic burdens and lower quality of life (6, 7). Although psychosocial function is influenced by many factors, there is growing evidence that social cognitive skills may be the key contributor (8–10). Social cognitive skills include the abilities to perceive, encode, process, and interpret social information (11, 12). Social cognition contains different domains, mainly involving social knowledge, social perception, theory of mind (ToM), attribution style, empathy, and emotion recognition (11, 13).

Among them, ToM and facial emotion recognition (FER) are two core structures that have been frequently studied. ToM is the ability to attribute mental states of other people [intentions, beliefs, and emotions]) (14). It is a complex construct with multiple components that is generally divided into two categories: cognitive and affective ToM. FER is the ability to identify a specific emotional state through the interpretation of another person's facial features (15–17).

To date, many studies have investigated impairments in ToM or FER in children and adolescents with epilepsy (18–21). However, most studies had small sample sizes and inconsistent conclusions. For example, some studies found that children and adolescents with epilepsy have significant ToM deficits compared to healthy controls (HCs) (20, 22–24), while others found no significant between-group differences (25, 26). Besides, for the recognition of anger, Pastorino et al. (20) found that children and adolescents with epilepsy exhibited large impairment compared to HCs, and Morningstar et al. (27) showed moderate impairment, whereas Wu et al. (21) and Braams et al. (28) found no significant differences between groups. Besides, the assessment methods used varied across studies (6, 19–22, 25, 29). A meta-analysis can improve statistical power and help refine the conclusions drawn from the inconsistent findings of previous studies.

To the best of our knowledge, six meta-analyses have examined the differences in social cognition between patients with epilepsy and HCs (30–35). However, one included only adult patients with epilepsy (34), the others included patients of different age groups (30–35). Furthermore, to date, no meta-analysis has examined social cognition performance in children with epilepsy or adolescents with epilepsy. Consequently, the primary aim of this study was to provide the first meta-analysis to examine differences between children and adolescents with epilepsy and HCs in terms of ToM and FER performance. Besides, subgroup analyses were performed to assess the impairment in different aspects of ToM (including cognitive ToM and affective ToM) and individual ToM tasks (such as strange stories test [SST]). In addition, taking into account appropriate control measures of ToM or FER tasks were also important factors (36), subgroup analyses were performed to assess the impairment in control measures of ToM or FER tasks (such as control measures of SST and control measures of FER). Moreover, considering that epileptic seizures were categorized by seizure onset into partial or generalized (37), subgroup analyses were conducted to examine social cognition deficits in children and adolescents with focal vs. generalized epilepsy. Additionally, given that temporal lobe epilepsy (TLE) and frontal lobe epilepsy (FLE) are more commonly studied in focal epilepsy (38, 39), subgroup analyses were also conducted to examine social cognition deficits in children and adolescents with TLE vs. FLE. Furthermore, meta-regression analyses were established to investigate whether the severity of ToM or FER impairment was moderated by potential demographic and epilepsy-related factors (such as sex, education level, age at testing, age at epilepsy onset, duration of epilepsy, full-scale Intelligence Quotient [IQ], verbal IQ, monthly seizure frequency, and number of anti-epileptic drugs [AEDs]). With this meta-analysis, we hope to promote a more comprehensive and nuanced understanding of how these two core domains of social cognition (ToM and FER) are affected in children and adolescents with epilepsy.

## Methods

### Study registration

This meta-analysis was conducted in line with the Preferred Reporting Items of Systematic Review and Meta-Analysis (PRISMA) guidelines (40). The protocol of this meta-analysis was registered at the International Platform of Registered Systematic Review and Meta-analysis Protocols (ID: INPLASY 202230011).

### Literature search strategy and data sources

Two investigators independently completed article retrieval, screening, quality assessment, and data extraction. Any disagreements were first discussed between the two investigators, and further disagreements were arbitrated by a third investigator.

A systematic literature search was conducted using Web of Science, PubMed, and Embase databases (up to July 21th, 2022). The following search terms were used: epilepsy or epileps^*^ or convulsion^*^ or seizure disorder combined with: social cognition or theory of mind or ToM or mentalizing or mentalising or Reading the Mind in the Eyes Test or Faux pas task or False Belief or the Awareness of Social Inference Test or Virtual Assessment of Mentalising Ability or the Movie for the Assessment of Social Cognition or picture sequencing task or Cartoon Test or Hinting Test or Strange Stories Test or sarcas^*^ or lie^*^ or joke^*^ or facial expression^*^ or facial emotion recognition. A backward citation search was also performed.

### Study selection

**Figure 1:**
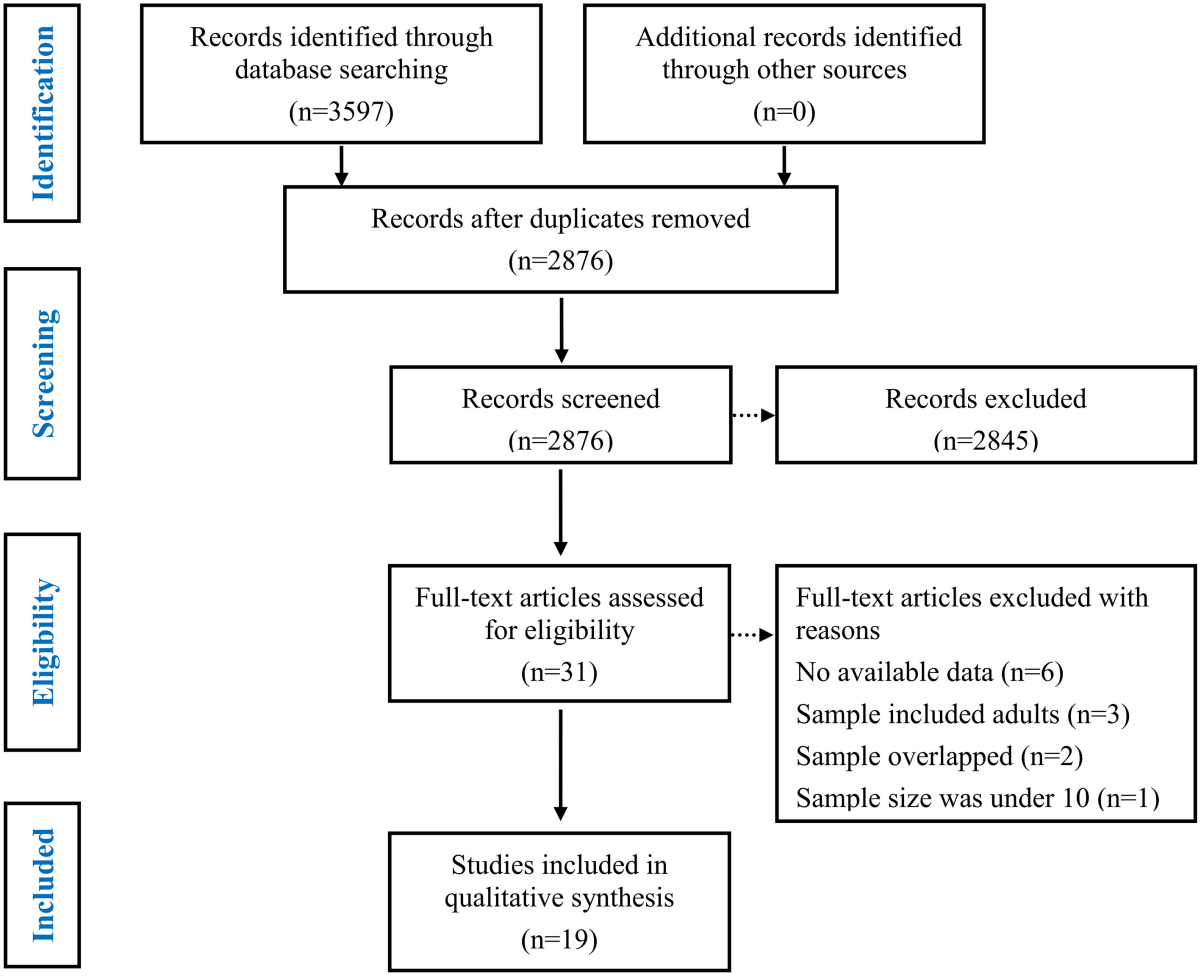
PRISMA flowchart of study screening and selection process.

First, duplicate items were removed. Subsequent primary screening of titles and abstracts were screened to remove ineligibility (i.e. literature reviews, abstracts, animal studies, no mention of epilepsy, or non-social cognition measures; see Figure 1).

Finally, a full-text screen was conducted.

Studies were included if they fit 5 criteria. First, they had to be published in peer-reviewed journals in English. Second, they included individuals with epilepsy < 18 years of age. Third, they compared children and adolescents with epilepsy to HCs group. Fourth, they assessed ToM or FER performance. Fifth, the statistics were published and could be used to calculate precise group comparison effect sizes. The authors were contacted if data were insufficient to calculate effect sizes. Studies were excluded if authors did not respond after 2 weeks. Studies were included if they provided data that could be used to calculate effect sizes for group comparisons.

Studies were excluded for 4 reasons. First, studies were excluded if they lacked a HCs group. Second, studies were excluded if they lacked comparisons between children and adolescents with epilepsy and HCs. Third, studies were excluded if the sample overlapped with another study with larger sample sizes. Fourth, studies were excluded if the sample size was <10 (41).

#### Study quality assessment

Study quality was assessed using a nine-star protocol based on the Newcastle-Ottawa-Scale (NOS) (42). For case-control studies, the NOS contains eight items grouped into three dimensions including selection, comparability, and exposure. Selection 1: Is the case definition adequate?; Selection 2: Representativeness of the cases; Selection 3: Selection of Controls; Selection 4: Definition of Controls; Comparability: Comparability of cases and controls on the basis of the design or analysis. Here, we selected “age at testing” as the most important adjusting factor and selected “sex” as other controlled factor; Exposure 1: Ascertainment of exposure; Exposure 2: Same method of ascertainment for cases and controls; Exposure 3: Non-Response rate. For each item a series of response options is provided. A maximum of one star each item is awarded for high-quality research, except for item related to comparability, where assignment of two stars is permitted. If star rating ≥ 7, the studies were considered high-quality (42).

#### Data extraction

The data included:

1) The basic title information mainly included name of first author, year of publication, and title.
2) Characteristics of the sample. The key variables here were number of participants in the epilepsy and control groups, epilepsy type, education level, age at testing, monthly seizure frequency, sex (female and male), number of AEDs, IQ score, and illness duration.
3) For ToM tasks, tasks were divided into cognitive and affective subcomponents.
4) Data used to calculate precise effect sizes for ToM or FER measures (e.g., mean and standard deviations, [SD] for the epilepsy group and HCs group).

#### Social cognition measures

Table 1 summarizes the different individual ToM tasks used, most common being the SST, faux pas task (FPT), and reading the mind in the eyes test (RMET); other tasks included false-belief test (FBT), ToM subscale from the second edition of the developmental neuropsychological assessment battery (NEPSY-II), Yoni task, ToM storybooks, ToM: Intentional lying, ToM: Sarcasm, ToM Inventory (TOMI).

**Table 1:** Characteristics and patient demographics of studies included in the meta-analysis.

**Study**	**Groups (female)**			**Epilepsy variables**								**ToM/FER task**
	**Epilepsy**	**HCs**	**Epilepsy type**	**Age (means, SD or range)**	**Education (years)**	**Full-scale IQ**	**Verbal IQ**	**Age at onset (years)**	**Duration (years)**	**Monthly seizure freq. (Per Month)**	**Number of AEDS**	
Bailey and Im-Bolter (43)	10 (3)	20 (9)	Generalized epilepsy	9.92 (2.34)	NA	NA	NA	3.59	NA	NA	NA	SST
Braams et al. (28)	41 (24)	82 (48)	Focal epilepsy	13.5 (4.4)	NA	NA	84	5.9	5.6	NA	NA	FER: FEEST
Braams et al. (23)	15 (10)	30 (20)	Focal epilepsy	7.1 (2.3)	NA	NA	80	3.8	3.5	NA	NA	ToM storybooks
Ciumas et al. (44)	13 (4)	11 (3)	Focal epilepsy	9.6 (1.7)	NA	NA	NA	7.54	1.99	NA	NA	FER: EDT
Esteso Orduña et al. (29)	22 (NA)	36 (NA)	Focal epilepsy (TLE)	NA	NA	NA	NA	5.3	4.09	NA	NA	FER: NEPSY-II
	23 (NA)		Focal epilepsy (FLE)	NA	NA	NA	NA	5.3	4.09	NA	NA	
Genizi et al. (25)	15 (NA)	15 (NA)	Focal epilepsy	10.53 (2.21)	NA	NA	NA	7.6	NA	NA	NA	Yoni task
Golouboff et al. (18)	29 (16)	37 (NA)	Focal epilepsy (TLE)	13.3 (2.9)	NA	96	97	5.4	6.5	NA	NA	FER: TREFE
	8 (5)		Focal epilepsy (FLE)	12.6 (2.7)	NA	101	105	5.8	4.7	NA	NA	
Lew et al. (45)	20 (12)	57 (29)	Generalized epilepsy	11.6 (2.5)	NA	95.1	NA	7.2	3.4	8.33	NA	SST, RMET
	27 (15)		Focal epilepsy	11.8 (2.17)	NA	87.5	NA	6.1	3.4	6.83	NA	
Lima et al. (24)	23 (8)	20 (5)	Focal epilepsy	11.2 (2.42)	5.48	100.43	NA	7.04	2.96	NA	NA	FPT
Lunn et al. (46)	56 (22)	62 (32)	Mixed epilepsy	11.66 (2.38)	NA	NA	NA	6.81	3.73	2.13	NA	SST, RMET
Morningstar et al. (27)	26 (9)	41 (26)	Focal epilepsy	14.15 (3.35)	NA	NA	NA	NA	NA	NA	NA	FER
Pastorino et al. (20)	62 (22)	60 (30)	Focal epilepsy	12.74 (3.4)	9.45	NA	NA	6.74	6	NA	1.6	ToM: NEPSY-II
												FER: NEPSY-II
Raud et al. (19)	35 (20)	30 (16)	Mixed epilepsy	10.46 (1.85)	NA	NA	NA	9.09	1.38	NA	NA	FBT, Intentional lying,
												Sarcasm
Stewart et al. (22)	22 (14)	22 (12)	Generalized epilepsy	12.82 (2.82)	NA	90.96	NA	6.36	6.18	13.24	1.41	SST, FPT, TOMI
Stewart et al. (17)	22 (11)	22 (12)	Focal epilepsy (TLE)	13.87 (2.21)	NA	NA	93.59	7.97	5.67	8.84	1.18	SST, FPT, TOMI
Stewart et al. (6)	22 (11)	22 (12)	Focal epilepsy (TLE)	13.87 (2.21)	NA	101.05	93.59	7.97	5.67	8.84	1.18	FER: POFA
	22 (14)		Generalized epilepsy	12.82 (2.82)	NA	90.96	NA	6.36	6.18	13.24	1.41	
Wu et al. (21)	33 (12)	33 (12)	Focal epilepsy	NA (8-10)	NA	NA	NA	NA	NA	NA	NA	FER: EBEDT
Zhang et al. (5)	54 (17)	37 (12)	Generalized epilepsy	11.94 (1.58)	6.19	NA	NA	8.79	3.31	NA	NA	FBT, FPT
Zilli et al. (26)	23 (11)	41 (NA)	Uncomplicated epilepsy	9.8 (2.6)	NA	104	NA	6.5	NA	NA	NA	ToM: NEPSY-II
												FER: NEPSY-II

NA, not available; HCs, healthy controls; SD, standard deviation; AEDS, antiepileptic drugs; ToM, theory of mind; IQ, Intelligence Quotient; FER, facial emotion recognition; TLE, temporal lobe epilepsy; FLE, frontal lobe epilepsy; SST, strange stories test; FEEST, facial expression of emotion stimuli and tests; EDT, emotion detection task; TREFE, the test de reconnaissance des emotions faciales pour enfants; RMET, reading the mind in the eyes test; FBT, false-belief test; TOMI, theory of mind inventory; POFA, the pictures of facial affect; EBEDT, the eye basic emotion discrimination task; FPT, faux pas test; NEPSY-II, the second edition of the developmental neuropsychological assessment battery.

Different FER tasks were used across studies, such as the FER subscale from NEPSY-II, facial expression of emotion stimuli and tests, emotion detection task, the test de reconnaissance des emotions faciales pour enfants, the pictures of facial affect, and the eye basic emotion discrimination task. Although definitions differed across tasks, all tasks required participants to identify and discriminate between the emotional states of others based on sets of photographs/drawings of basic facial emotions.

#### Cognitive ToM and affective ToM

Cognitive ToM involves the cognitive understanding of the other person's thoughts, intentions, and beliefs (47–49). It can be evaluated through several tasks such as the SST, ToM storybooks, FBT, and TOMI, as well as the cognitive subcomponents of the Yoni task, FPT, and ToM subscale from NEPSY-II.

Affective ToM is described as the capacity to infer another person's emotional states (47–49). It can be evaluated through several tasks such as the RMET, ToM: Intentional lying, and ToM: Sarcasm, as well as the affective subcomponents of the Yoni task, FPT, and ToM subscale from NEPSY-II.

### Statistical analysis

For analyses, the Stata 15.0 software package was used, with Hedges *g* the index of effect size (50, 51), and effects of 0.2 interpreted as small, 0.5 indicates a medium effect, and values equal to or larger than 0.8 indicates a large effect (52). Meta-analyses were completed using a random-effects model. Negative effect sizes values indicated poorer performance for children and adolescents with epilepsy compared to HCs.

For studies that did not provide total scores on overall ToM performance, but reported multiple individual ToM tasks, pooled effect sizes were aggregated by calculating the mean effect size and standard error (53). Aggregate effect sizes for cognitive ToM, affective ToM, or overall FER were calculated in a similar manner.

We used I^2^ statistics to assess study heterogeneity. As suggested by Higgins, values of 0–25% indicate no heterogeneity, I^2^ values between 25 and 50% indicate small magnitudes of heterogeneity, and I^2^ values in the range 50 and 75% are interpreted as moderate heterogeneity, while >75% are explained for large heterogeneity (54, 55). To assess risk of publication bias, the Egger's test was evaluated (56). In the case of a significant Egger's test, the trim-and-fill method was applied to provide adjusted effect sizes (57).

Subgroup analysis were conducted for different aspects of ToM (including cognitive ToM and affective ToM), individual ToM tasks (including SST, FPT, RMET, FBT, and ToM subscale from NEPSY-II), control measures of ToM tasks (including control measures of SST, control measures of FPT, and control measures of FBT), and individual emotions recognition (including happy, anger, fear, sad, disgust, neutral, and surprise). Besides, subgroup analyses were also conducted to examine social cognition deficits in children and adolescents with focal vs. generalized epilepsy, and in children and adolescents with TLE vs. FLE (more information is provided in Supplementary Table 1).

In addition, of the included studies, 12 of the 19 studies explicitly limited their samples to individuals with an IQ score >70 (5, 6, 17–22, 24–27, 43). Although the other 4 studies did not explicitly limit IQ scores in the exclusion/inclusion criteria, the mean IQ scores of the samples were significantly >70 (23, 28, 29, 45). The other two included children with benign childhood epilepsy with centrotemporal spikes (BECTS) (25, 44), who usually exhibited normal intelligence (58–60). The remaining 1 study limited samples to individuals with an IQ score >60, and of the 56 patients included, 6 were in the range of 60 to 69 and the others ≥70 (46). Considering that the IQ of the included sample may impact the results (60), subgroup analysis were conducted to examine social cognition deficits in samples with an IQ score >70.

Meta-regression analyses were conducted to investigate whether the severity of overall ToM or overall FER impairment was affected by potential demographic and epilepsy-related factors (sex, education level, age at testing, age at epilepsy onset, duration of epilepsy, full-scale IQ, verbal IQ, monthly seizure frequency, and number of AEDs). The significance level was set at *p* < 0.05. For each individual factors in the meta-regression model, a minimum of 3 data points was required (61).

## Results

### Study characteristics

The study selection process is summarized in Figure 1. In total, 3643 potentially eligible articles were retrieved. After the removal of duplicates, 2904 articles remained, which were then subjected to title and abstract screening. Of these, 31 initially met the inclusion criteria. After full-text screening, 12 were excluded for the following reasons: the study did not provide sufficient data to calculate the effect sizes of ToM or FER (the number of studies [*k*] = 6) (62–67); the study population included adults (*k* = 3) (68–70); the samples overlapped with those of other studies (*k* = 2) (71, 72); and the sample size was under 10 (*k* = 1) (73). Finally, a total of 19 studies with 623 children and adolescents with epilepsy (mean age = 12.13 years, SD = 2.62, 46.1 % female) and 677 HCs (mean age = 11.48 years, SD = 2.71, 50.7 % female) were included in this meta-analysis (Table 1) (5, 6, 17–29, 43–46). Of these studies included, 12 studies (384 children and adolescents with epilepsy and 473 HCs) examined overall ToM and 9 datasets examining FER (324 children and adolescents with epilepsy and 459 HCs).

The mean score of the study quality assessment was 7.21 (SD = 0.92), and 15 of the 19 case-control studies were awarded ≥ 7 stars and considered of high quality (Table 2).

**Table 2:** Quality evaluation of included studies.

**Study**	**S1**	**S2**	**S3**	**S4**	**C**	**E1**	**E2**	**E3**	**Sum**
Bailey and Im-Bolter (43)	⋆	—	—	⋆	⋆⋆	⋆	⋆	⋆	7
Braams et al. (28)	⋆	⋆	—	⋆	⋆⋆	⋆	⋆	⋆	8
Braams et al. (23)	⋆	⋆	—	⋆	⋆⋆	⋆	⋆	⋆	8
Ciumas et al. (44)	⋆	—	—	⋆	⋆⋆	⋆	⋆	⋆	7
Esteso Orduña et al. (29)	⋆	—	—	⋆	— —	⋆	⋆	⋆	5
Genizi et al. (25)	⋆	—	—	⋆	⋆ —	⋆	⋆	⋆	6
Golouboff et al. (18)	⋆	—	—	⋆	⋆⋆	⋆	⋆	⋆	7
Lew et al. (45)	⋆	⋆	—	⋆	⋆⋆	⋆	⋆	⋆	8
Lima et al. (24)	⋆	—	—	⋆	⋆⋆	⋆	⋆	⋆	7
Lunn et al. (46)	⋆	—	—	⋆	⋆ —	⋆	⋆	⋆	6
Morningstar et al. (27)	⋆	—	—	⋆	⋆ —	⋆	⋆	⋆	6
Pastorino et al. (20)	⋆	⋆	—	⋆	⋆⋆	⋆	⋆	⋆	8
Raud et al. (19)	⋆	—	—	⋆	⋆⋆	⋆	⋆	⋆	7
Stewart et al. (22)	⋆	⋆	—	⋆	⋆⋆	⋆	⋆	⋆	8
Stewart et al. (17)	⋆	⋆	—	⋆	⋆⋆	⋆	⋆	⋆	8
Stewart et al. (6)	⋆	⋆	—	⋆	⋆⋆	⋆	⋆	⋆	8
Wu et al. (21)	⋆	⋆	—	⋆	⋆⋆	⋆	⋆	⋆	8
Zhang et al. (5)	⋆	—	⋆	⋆	⋆⋆	⋆	⋆	⋆	8
Zilli et al. (26)	⋆	—	—	⋆	⋆⋆	⋆	⋆	⋆	7

We herein selected “age at testing” as the most important adjusting factor and selected “sex” as other controlled factor. S1: Is the case definition adequate?; S2: Representativeness of the cases; S3: Selection of Controls; S4: Definition of Controls; C: Comparability of cases and controls on the basis of the design or analysis; E1: Ascertainment of exposure; E2: Same method of ascertainment for cases and controls; E3: Non-Response rate. Each star (⋆) represents one point.

### ToM in children and adolescents with epilepsy

**Figure 2:**
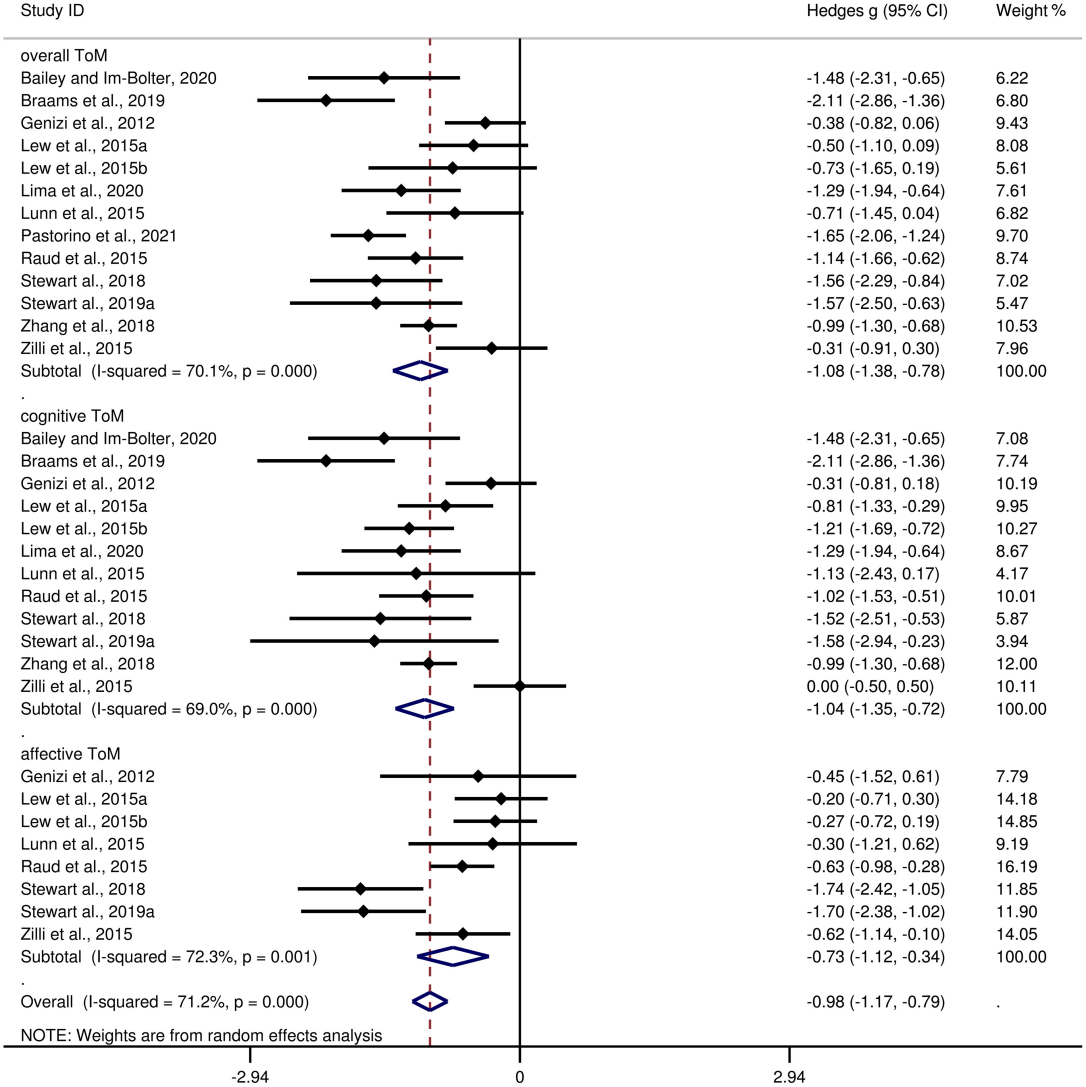
Forest plots showing effect size estimates for overall ToM, cognitive ToM, and affective ToM differences between children and adolescents with epilepsy and healthy controls.

Table 3 shows the relevant results of this meta-analysis. Compared to HCs, children and adolescents with epilepsy had impairment in overall ToM, with this deficit being significant in the magnitude (*g* = −1.08, 95% *CI* [−1.38, −0.78], *z* = −7.10, *p* < 0.001, *k* = 13, see Figure 2). When considering the different subcomponents of ToM, the findings showed that children and adolescents with epilepsy was associated with large impairment in cognitive ToM (*g* = −1.04, 95% *CI* [−1.35, −0.72], *z* = −6.38, *p* < 0.001, *k* = 12, see Figure 2) and medium impairment in affective ToM (*g* = −0.73, 95% *CI* [−1.12, −0.34], *z* = −3.65, *p* < 0.001, *k* = 8, see Figure 2). For individual ToM tasks (Supplementary Figure 1), children and adolescents with epilepsy performed worse than the HCs on SST, FPT, and FBT measures. However, no group differences were observed for performance as indicated by RMET or ToM subscale from NEPSY-II measures. Besides, for control measures of ToM tasks (Supplementary Figure 2), relative to the HCs, children and adolescents with epilepsy performed worse on control measures of SST and FPT, but no significant differences were observed for control measures of FBT. For studies that included only samples with an IQ score >70, relative to HCs, children and adolescents with epilepsy exhibited large impairments in overall ToM and cognitive ToM, and medium impairment in affective ToM.

**Table 3:** Mean effects for ToM and FER subcomponents comparing children and adolescents with epilepsy against healthy controls and tests for publication bias.

**Test**	***k***	***n* in epilepsy group**	***n* in HCs group**	***g***	**95%** ***CI***				**Test for heterogeneity**	**Assess risk of publication bias**
					**Lower**	**Upper**	***z* value**	***p* value**	**I^2^ Statistic, %**	**Egger's test *p* value**
Overall ToM	13	384	473	−1.08	−1.38	−0.78	−7.10	<0.001	70	0.634
Overall ToM^*^	12	328	411	−1.11	−1.43	−0.80	−6.89	<0.001	72	0.550
Cognitive ToM	12	322	413	−1.04	−1.35	−0.72	−6.38	<0.001	69	0.242
Cognitive ToM^*^	11	266	351	−1.03	−1.37	−0.70	−6.11	<0.001	72	0.239
Affective ToM	8	220	306	−0.73	−1.12	−0.34	−3.65	<0.001	72	0.495
Affective ToM^*^	7	164	244	−0.77	−1.20	−0.35	−3.58	<0.001	76	0.348
SST	6	157	240	−1.70	−2.40	−0.99	−4.71	<0.001	82	0.278
FPT	4	121	101	−1.27	−1.54	−1.00	−9.30	<0.001	0	0.788
RMET	3	103	176	−0.25	−0.56	0.07	−1.52	0.129	0	0.958
FBT	2	89	67	−0.99	−1.32	−0.66	−5.82	<0.001	0	
ToM subscale from NEPSY-II	2	85	101	−1.00	−2.31	0.31	−1.50	0.135	92	
Control measures of SST	6	157	240	−0.41	−0.65	−0.16	−3.29	0.001	0	0.593
Control measures of FPT	3	98	81	−0.47	−0.88	−0.07	−2.29	0.022	43	0.505
Control measures of FBT	1	54	37	−0.27	−0.69	0.15	−1.27	0.206	0	
Overall FER	12	324	459	−0.98	−1.33	−0.64	−5.62	<0.001	83	0.193
Happy	9	256	346	−0.19	−0.38	0.01	−1.91	0.056	26	0.436
Anger	8	243	335	−0.45	−0.75	−0.15	−2.94	0.003	67	0.933
Fear	9	256	346	−0.49	−0.82	−0.15	−2.86	0.004	71	0.564
Sad	8	243	335	−0.78	−0.98	−0.57	−7.45	<0.001	27	0.569
Disgust	7	217	293	−0.71	−0.96	−0.46	−5.56	<0.001	44	0.089
Neutral	6	169	220	−0.72	−0.95	−0.49	−6.23	<0.001	0	0.818
Surprise	2	74	115	−0.39	−1.13	0.35	−1.04	0.300	83	

HCs, healthy controls; CI, confidence interval; FER, facial emotion recognition; k, the number of studies; SST, strange stories test; ToM, theory of mind; FPT, faux pas test; RMET, reading the mind in the ryes test; FBT, false-belief test; NEPSY-II, the second edition of the developmental neuropsychological assessment battery; n, the number; g, Hedges g;

* : the effect size of studies that included only samples with an Intelligence Quotient score >70.

There was no heterogeneity across studies for FPT, FBT, RMET, control measures of SST, or control measures of FBT (I^2^ = 0). Small heterogeneity was found for control measures of FPT (I^2^ = 43%), moderate heterogeneity was found for overall ToM (I^2^ = 70%), cognitive ToM (I^2^ = 69%), and affective ToM (I^2^ = 72%), and significant heterogeneity was found among studies on SST (I^2^ = 82%) and NEPSY-II (I^2^ = 92%). There was no significant publication bias for overall ToM, cognitive ToM, affective ToM, any individual ToM tasks, or any control measures of ToM tasks.

### Meta-regression analysis for overall ToM

The following variables did not account for the significant variance in overall ToM (sex, *t* = −0.18, *p* = 0.857, *k* =12; age at testing, *t* = 0.14, *p* = 0.892, *k* =13; age at epilepsy onset, *t* =1.4, *p* = 0.190, *k* =13; duration of epilepsy, *t* = −1.41, *p* =0.195, *k* =10; full-scale IQ, *t* = 0.66, *p* =0.554, *k* =5; education level, *t* = −1.76, *p* =0.328, *k* =3; monthly seizure frequency, *t* = −1.34, *p* = 0.274, *k* =5; number of AEDs, *t* = −0.21, *p* = 0.886, *k* =3). As fewer than 3 studies contributed to the data for this subcomponent, no meta-regression analysis was performed for the effect of verbal IQ on the severity of overall ToM.

### FER in children and adolescents with epilepsy

**Figure 3:**
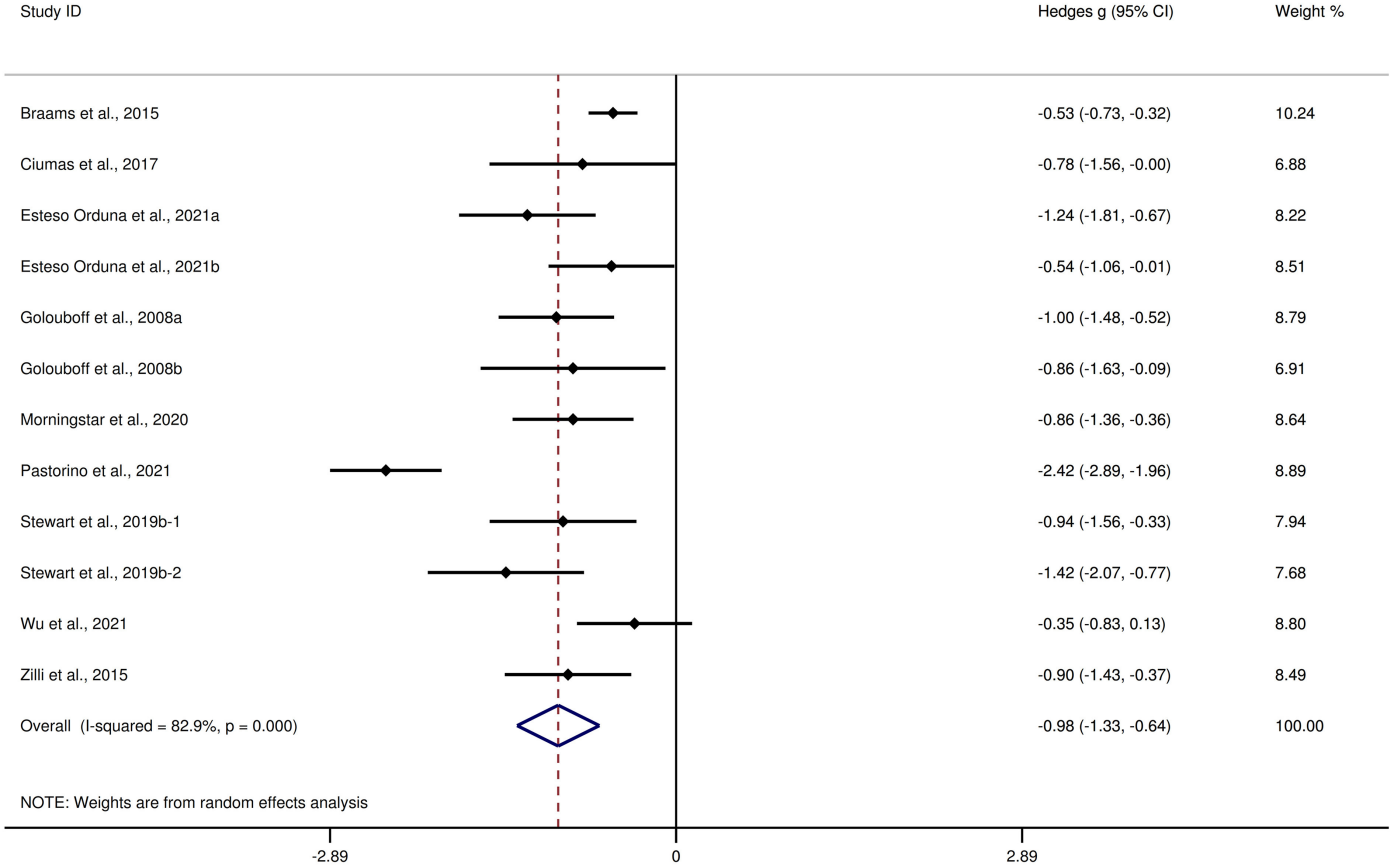
Forest plots showing effect size estimates for FER differences between children and adolescents with epilepsy and healthy controls.

Table 3 shows the significant results of this meta-analysis. For overall FER, children and adolescents with epilepsy exhibited a large impairment compared to HCs (*g* = −0.98, 95% *CI* [−1.33, −0.64], *z* = −5.62, *p* < 0.001, *k* = 12, see Figure 3). For the analyses of individual emotions (Supplementary Figure 3), epilepsy in children and adolescents was associated with medium impairments in sad, disgust, and neutral recognition, and small impairments in anger and fear recognition. However, no group differences were evident for happy or surprise. As the included studies did not report control measures of FER tasks, we did not perform an analysis of the measures of FER tasks here.

There was no heterogeneity across studies for neutral, small heterogeneity for happy, sad, and disgust (I^2^ = 26%, I^2^ = 27%, and I^2^ = 44%, respectively), moderate heterogeneity for anger and fear (I^2^ = 67% and I^2^ = 71%, respectively), and significant heterogeneity for studies on overall FER (I^2^ = 83%) and surprise (I^2^ = 83%). There was no significant publication bias for overall FER or any individual emotions.

### Meta-regression analysis for FER

The variables did not account for the significant variance in the overall FER (sex, *t* = −0.64, *p* = 0.543, *k* = 10; age at testing, *t* = 0.31, *p* = 0.764, *k* = 9; age at epilepsy onset, *t* = −0.22, *p* = 0.832, *k* = 10; duration of epilepsy, *t* = −0.37, *p* = 0.721, *k* = 9; full-scale IQ, *t* = 1.18, *p* = 0.322, *k* = 5; verbal IQ, *t* = −1.94, *p* = 0.192, *k* = 4; number of AEDs, *t* = 0.27, *p* = 0.83, *k* = 3). As fewer than 3 studies contributed to the data for this subcomponent, no meta-regression analysis was performed for the effect of education level or monthly seizure frequency on the severity of overall FER.

### ToM and FER in children and adolescents with focal epilepsy and generalized epilepsy

**Figure 4:**
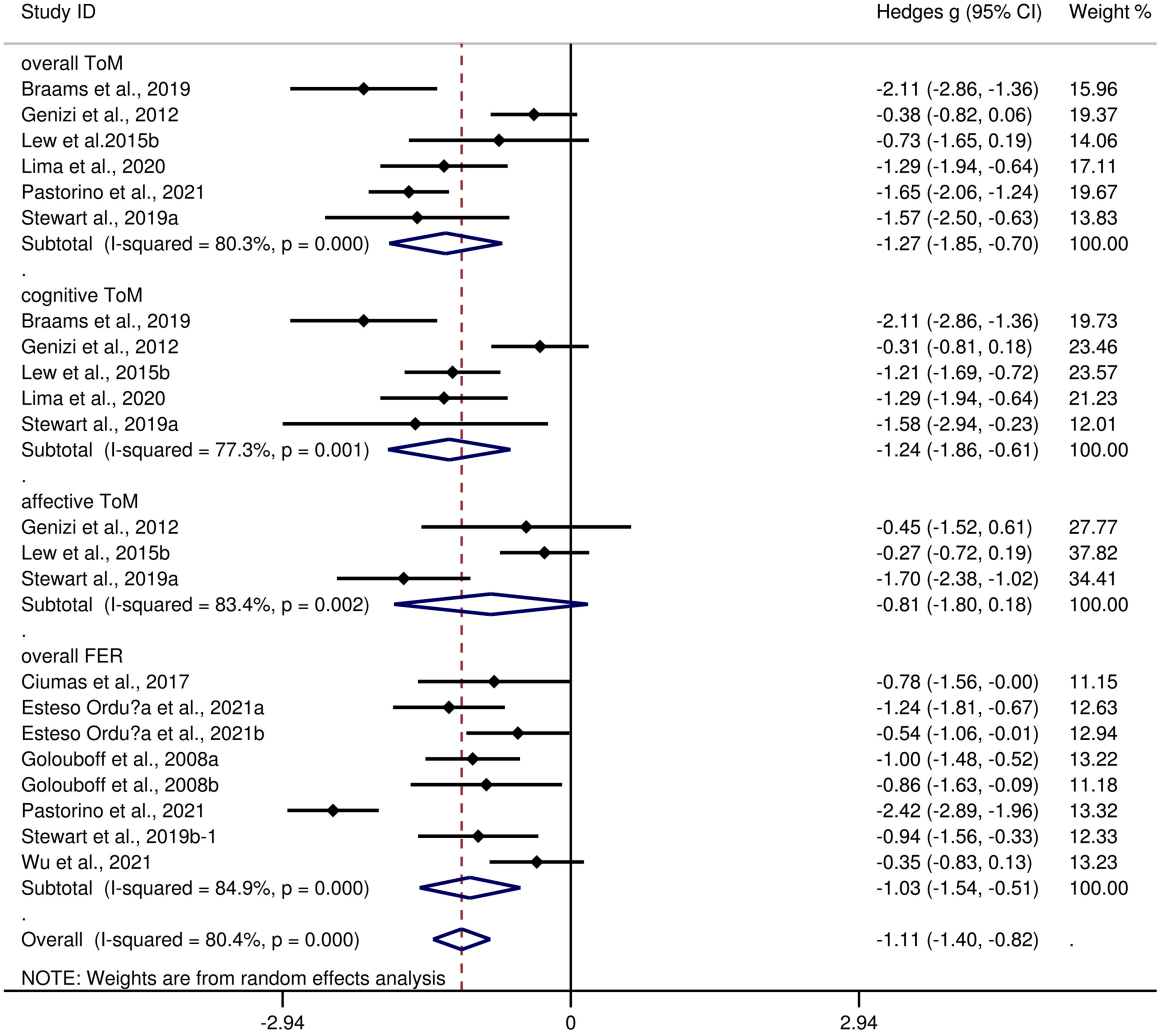
Forest plots showing effect size estimates for o overall ToM, cognitive ToM, affective ToM, and overall FER differences between focal epilepsy and healthy controls.

**Figure 5:**
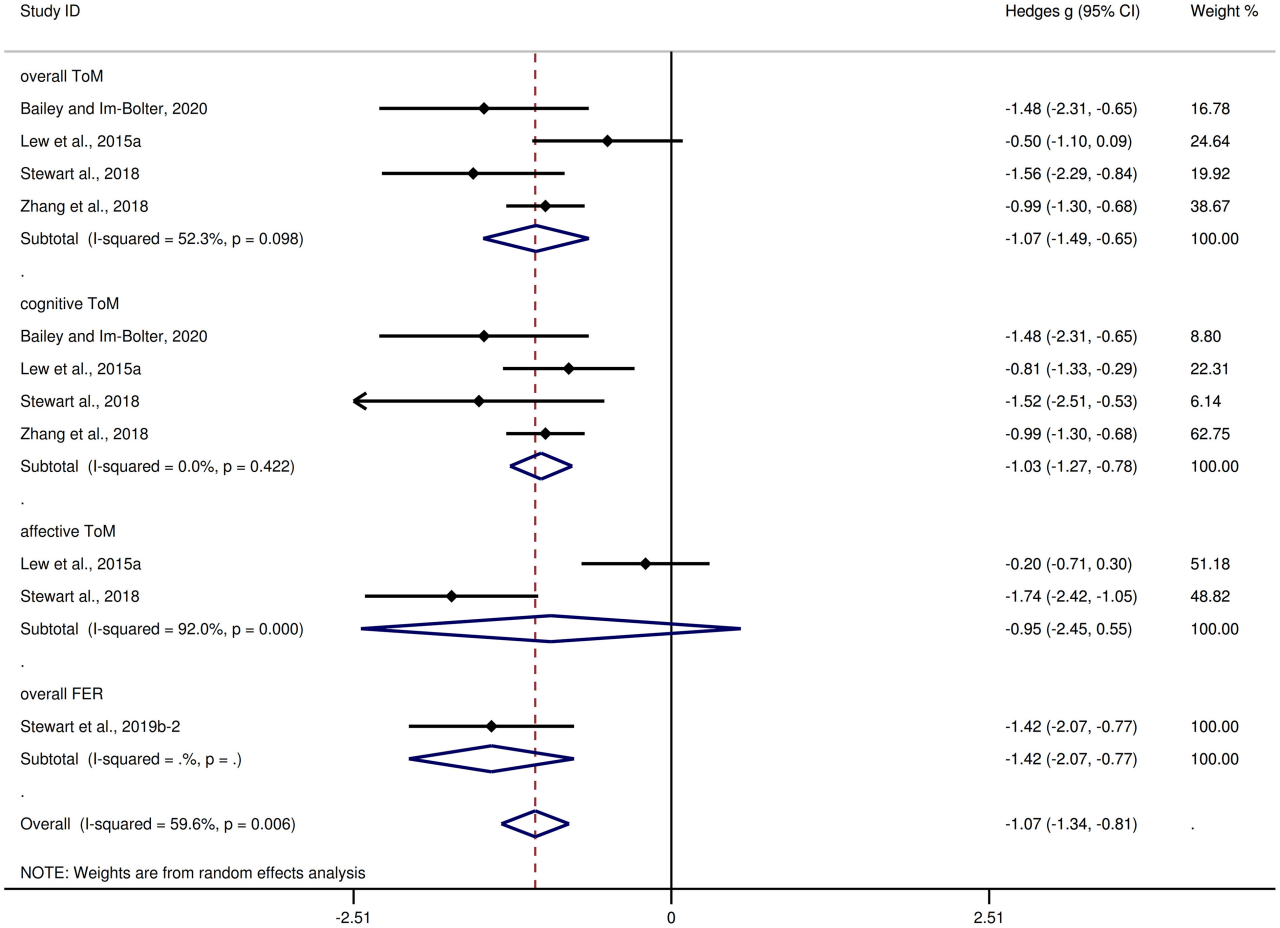
Forest plots showing effect size estimates for overall ToM, cognitive ToM, affective ToM, and overall FER differences between generalized epilepsy and healthy controls.

Table 4 depicts the key results obtained from this meta-analysis. Subgroup analyses revealed that the performance of children and adolescents with focal epilepsy (Figure 4) and generalized epilepsy (Figure 5) with respect to overall ToM (*g* = −1.27, 95% *CI* [−1.85, −0.70], *z* = −4.34, *p* < 0.001, *k* = 6 and *g* = −1.07, 95% *CI* [−1.49, −0.65], *z* = −5.01, *p* < 0.001, *k* = 4, respectively), cognitive ToM (*g* = −1.24, 95% *CI* [−1.86, −0.61], *z* = −3.89, *p* < 0.001, *k* = 5 and *g* = −1.03, 95% *CI* [−1.27, −0.78], *z* = −8.21, *p* < 0.001, *k* = 4, respectively),and overall FER (*g* = −1.03, 95% *CI* [−1.54, −0.51], *z* = −3.92, *p* < 0.001, *k* = 8 and *g* = −1.42, 95% *CI* [−2.07, −0.77], *z* = −4.27, *p* < 0.001, *k* = 1, respectively) was inferior to that of the HCs. In affective ToM, children and adolescents with focal epilepsy and generalized epilepsy did not differ from HCs (*g* = −0.81, 95% *CI* [−1.80, 0.18], *z* = −1.61, *p* = 0.107, *k* = 3 and *g* = −0.95, 95% *CI* [−2.45, 0.55], *z* = −1.24, *p* = 0.214, *k* = 2, respectively).

**Table 4:** Mean effects for ToM and FER subcomponents comparing focal epilepsy and generalized epilepsy against healthy controls and tests for publication bias.

**Test**	***k***	***n* in focal epilepsy group**	***N* In Hcs group**	***g***	**95%** ***CI***				**Test for heterogeneity**	**Assess risk of publication bias**
					**Lower**	**Upper**	***z* value**	***p* value**	**I^2^ Statistic,%**	**Egger's test *p* value**
Overall ToM	6	164	204	−1.27	−1.85	−0.70	−4.34	<0.001	80	0.714
Cognitive ToM	5	102	144	−1.24	−1.86	−0.61	−3.89	<0.001	77	0.352
Affective ToM	3	64	94	−0.81	−1.80	0.18	−1.61	0.107	83	0.75
Overall FER	8	212	272	−1.03	−1.54	−0.51	−3.92	<0.001	85	0.552
		*n* in generalized epilepsy group	*n* in HCs group							
Overall ToM	4	106	136	−1.07	−1.49	−0.65	−5.01	<0.001	52	0.637
Cognitive ToM	4	106	136	−1.03	−1.27	−0.78	−8.21	<0.001	0	0.297
Affective ToM	2	42	79	−0.95	−2.45	0.55	−1.24	0.214	92	
Overall FER	1	22	22	−1.42	−2.07	−0.77	−4.27	<0.001		

HCs, healthy controls; CI, confidence interval; FER, facial emotion recognition; g, Hedges g; ToM, theory of mind; n, the number; k, the number of studies.

The effect sizes of the focal epilepsy and generalized epilepsy groups were comparable for overall ToM (*Q* = 0.32, *df* = 1, *p* = 0.570), cognitive ToM (*Q* = 0.38, *df* = 1, *p* = 0.538), affective ToM (*Q* = 0.02, *df* = 1, *p* = 0.880), and overall FER (*Q* = 0.86, *df* = 1, *p* = 0.353).

### ToM and FER in children and adolescents with TLE and FLE

Supplementary Table 1 depicts the key results obtained from this meta-analysis. Compared to HCs, children and adolescents with TLE had large impairment in overall ToM (*g* = −1.56, 95% *CI* [−2.50, −0.63], *z* = −3.27, *p* = 0.001, *k* = 1), cognitive ToM (*g* = −1.58, 95% *CI* [−2.94, −0.23], *z* = −2.30, *p* = 0.022, *k* = 1), and affective ToM (*g* = −1.70, 95% *CI* [−2.38, −1.02], *z* = −4.91, *p* < 0.001, *k* = 1). For overall FER, relative to HCs, children and adolescents with TLE showed large impairment (*g* = −1.06, 95% *CI* [−1.37, −0.74], *z* = −6.58, *p* < 0.001, *k* = 3), while children and adolescents with FLE showed moderate impairment (*g* = −0.64, 95% *CI* [−1.08, −0.21], *z* = −2.89, *p* = 0.004, *k* = 2).

The effect sizes of the TLE and FLE groups were comparable overall FER (*Q* = 2.398, *df* = 1, *p* = 0.121).

## Discussion

To our knowledge, this is the first meta-analysis to investigate the patterns of ToM and FER function in children and adolescents with epilepsy. It included 19 studies, with a combined total sample size of 623 children and adolescents with epilepsy and 677 HCs. The results showed that, relative to HCs, children and adolescents with epilepsy exhibited large impairments in overall ToM, cognitive ToM, overall FER (*g* = −1.08, *g* = −1.04, and *g* = −0.98, respectively), and medium impairment in affective ToM (*g* = −0.73). The subgroup analyses found no statistically significant differences in the degree of ToM or FER impairments between children and adolescents with focal epilepsy and generalized epilepsy. Besides, the degree of FER impairment was not statistically different between children and adolescents with TLE and FLE. Furthermore, in individual ToM tasks, compared to the HCs, children and adolescents with epilepsy exhibited impairments in SST, FPT, and FBT, but no significant differences were observed for RMET or ToM subscale from NEPSY-II. Besides, in control measures of ToM tasks, relative to the HCs, children and adolescents with epilepsy exhibited impairments in control measures of SST and FPT, but no significant differences were observed for control measures of FBT. Additionally, for studies that included only samples with an IQ score >70, relative to HCs, children and adolescents with epilepsy exhibited large impairments in overall ToM and cognitive ToM, and medium impairment in affective ToM. Regarding individual emotions, children and adolescents with epilepsy were associated with the medium impairments in sad, disgust, and neutral recognition, and small impairments in anger and fear recognition. However, no group differences were evident for happy or surprise recognition. The meta-regression analyses indicated that the variables (sex, education level, age at testing, age at epilepsy onset, duration of epilepsy, full-scale IQ, verbal IQ, monthly seizure frequency, and number of AEDs) did not affect the magnitude of the effect sizes observed.

Our meta-analysis showed significant overall ToM dysfunction in children and adolescents with epilepsy (*g* = −1.08, *k* = 13). The results support the findings of Eicher and Jokeit (33) (*g* = −0.87, *k* = 26), who conducted a meta-analysis to report differences in the overall ToM between patients with epilepsy (including different epilepsy phenotypes and different age groups) and the HCs. However, it was different from the findings of Stewart et al. (31) and Wang et al. (34) (*g* = −0.68, *k* = 45 and *g* = −0.73, *k* = 12, respectively), which indicate a moderate-sized impairment. In terms of the subcomponents of the ToM, children and adolescents with epilepsy had a large impairment in cognitive ToM and a medium impairment in affective ToM. This finding confirms the previous consensus that the cognitive and affective parts of ToM are partly separate skills, relying on common and distinct neural networks (74–77). Specifically, they involve a common network of brain regions, including the superior temporal sulcus, bilateral temporal poles, medial prefrontal cortex, and temporo-parietal junction (78, 79). In addition to activation of a common network, cognitive ToM and affective ToM are associated with greater activation in the dorsolateral (80) and ventromedial (74, 81), prefrontal cortices, respectively. Coincidentally, these areas, especially the dorsolateral prefrontal cortex, appear to be more susceptible to impairment in children and adolescents with epilepsy (82–85).

Similar to ToM, our meta-analysis showed that children and adolescents with epilepsy had significant FER dysfunction (*g* = −0.98, *k* = 12). The result was consistent with the findings of Edwards et al. (32), who conducted a meta-analysis of 24 studies and reported a difference in the overall FER (*g* = −0.99) between patients with epilepsy and the HCs. In reference to the individual emotions, children and adolescents with epilepsy had small effect sizes for the recognition of fear and anger, and medium impairment in sad, disgust, and neutral, while no differences were observed for the recognition of happy or surprise. These results support the conclusions of previous qualitative studies indicating that children and adolescents with epilepsy have marked deficits in recognizing negative emotional states, but have little difficulty recognizing positive emotional states (18, 21, 71, 86, 87). Among negative emotional states, recognition defect of fear emotion is the most common in epilepsy, which may be related to structural and functional abnormalities in the insular cortex, dorsal striatum, and amygdala (20, 21, 88–91). It has been reported that found that deficits in recognizing fear may be related to psychosocial adjustment difficulties in children with epilepsy (18). Similar relationships have been observed in previous psychopathological studies for children and adolescents: impaired fear recognition correlated with externalizing and internalizing problems (92–96). In addition, the impairment of recognition of anger, sad, disgust, and neutral in children and adolescents with epilepsy may be associated with the abnormalities in the structure and function of the orbital frontal cortex, anterior insula, and somatosensory cortices (97–100). The relatively intact recognition of happy is not unique to children and adolescents with epilepsy, but is common in a variety of neurodegenerative diseases (13, 101–103). This may be since happy, the first emotion humans recognize, is easier to be identified than other emotions (71, 104, 105). Furthermore, the relatively intact recognition of surprise may be because surprise, as one positive emotion, is a common and basic facial expression (100). This differential recognition of individual emotions is consistent with previous findings that different types of neural dysfunction may present different emotional recognition impairments (13, 101, 106).

In the subgroup meta-analyses, no statistically significant differences were found in the degree of ToM or FER impairment between children and adolescents with focal epilepsy and generalized epilepsy or the degree of FER impairment between children and adolescents with TLE and FLE. These results of this study support previous reports that children and adolescents with focal and generalized epilepsy may experience roughly equivalent social cognitive impairments (107). This is consistent with the understanding of epilepsy as a network disease revised by the International League Against Epilepsy (37, 71, 108). Specifically, seizures arise from a shared neural network involving overlapping cortical and subcortical structures, regardless of whether seizures originate from an identified pathological site (37, 108, 109). In addition, our study showed no difference in affective ToM between children and adolescents with focal epilepsy or generalized epilepsy and HCs. However, it should be noted the fact that different focal seizures were grouped together in this analysis. Considering that focal epilepsy contains multiple epilepsy phenotypes, and that the pathophysiology of different epilepsy phenotypes is different (110), we should interpret the conclusions of focal epilepsy vs. generalized epilepsy with caution. In addition, although we performed an analysis of TLE vs. FLE commonly seen in focal epilepsy, however, due to the limited number of studies included (*k* = 3 and *k* = 2 in this study), one must conclude cautiously.

From a therapeutic perspective, interventions targeting social cognition may be an effective approach to address social difficulties in children and adolescents with epilepsy (107). Currently, only one research protocol has been reported outlining a novel cognitive-behavioral intervention using ToM training specifically for children with epilepsy (111). In addition, in children and adolescents with other neurodevelopmental disorders, such as autism spectrum disorders, and typically developing children with social handicaps, social function and social cognition were significantly improved through social cognitive interventions (112–117). These findings suggest that social cognitive therapy holds promise for children and adolescents with epilepsy. These quantitative results further deepen our understanding of the two core domains of social cognition in children and adolescents with epilepsy and may assist in the development of cognitive interventions for this patient population.

## Limitations

Our findings have to take into account certain limitations. First, although 19 studies were included in this meta-analysis, none investigated ToM performance in children and adolescents with FLE, and only 1 study specifically investigated ToM performance in children and adolescents with TLE. In addition, there are few studies available for some individual ToM tasks or individual emotions, such as NEPSY-II, FBT, and recognition of surprise (*k* = 2, respectively). Therefore, more research is needed in the future to solidify conclusions. Second, the current meta-analysis is a cross-sectional study. Longitudinal studies are necessary to further evaluate the dynamic changes of social cognition function in children and adolescents with epilepsy. Third, although we investigated the potential effects of some factors (sex, age at testing, age at epilepsy onset, duration of epilepsy, full-scale IQ, verbal IQ, education level, monthly seizure frequency, and number of AEDs) on social cognition function, we should interpret the results with caution due to the lack of literature, such as the association of education level or number of AEDs on ToM performance (*k* = 3, respectively). In addition, due to the insufficient data included, other factors (such as functional impairment, depression, and neuropsychiatric symptoms) were not examined. Therefore, more research is needed in these aspects in the future. Fourth, social cognition contains different domains, of which the most researched are ToM, FER, and empathy. Our study only examined ToM and FER patterns in children and adolescents with epilepsy. It is necessary to assess empathy performance in children and adolescents with epilepsy in the future.

## Conclusions

In conclusion, this is the first comprehensive meta-analysis examining the patterns of ToM and FER in children and adolescents with epilepsy. This quantitative result showed that children and adolescents with epilepsy exhibited deficits in two key domains of social cognition (ToM and FER) and ToM subcomponents (cognitive and affective ToM). In addition, children and adolescents with focal epilepsy and generalized epilepsy had no statistically significant differences in social cognition deficits. Future studies investigating the neural correlates of social cognition deficits in children and adolescents with epilepsy and longitudinal studies are needed, which may further reveal the nature and course of social cognition impairment in children and adolescents with epilepsy.

## Data availability statement

The original contributions presented in the study are included in the article/Supplementary material, further inquiries can be directed to the corresponding authors.

## Author contributions

ZQY and LLX designed this study. YS, JZ, PWZ, HZ, and JGZ analyzed and interpreted relevant data. YS and ZQY drafted the manuscript. ZQY and LLX revised the manuscript. All authors have read and approved the final manuscript.

## Funding

This research was supported by Jiangsu Commission of Health (LGY20180390).

## Conflict of interest

The authors declare that the research was conducted in the absence of any commercial or financial relationships that could be construed as a potential conflict of interest.

## Publisher's note

All claims expressed in this article are solely those of the authors and do not necessarily represent those of their affiliated organizations, or those of the publisher, the editors and the reviewers. Any product that may be evaluated in this article, or claim that may be made by its manufacturer, is not guaranteed or endorsed by the publisher.

## Supplementary material

Supplementary Figure 1 Forest plots showing effect size estimates for individual ToM tasks differences between children and adolescents with epilepsy and healthy controls.

Supplementary Figure 2 Forest plots showing effect size estimates for control measures of ToM tasks differences between children and adolescents with epilepsy and healthy controls.

Supplementary Figure 3 Forest plots showing effect size estimates for individual emotions differences between children and adolescents with epilepsy and healthy controls.

Supplementary Table 1 Mean effects for ToM and FER subcomponents comparing TLE and FLE against healthy controls and tests for publication bias.
