# Supplementary figures and images for: Social cognition in children and adolescents with epilepsy: A meta-analysis
Source: Front Psychiatry. 2022 Sep 15;13:983565. doi: 10.3389/fpsyt.2022.983565 (PMC9520261; doi:10.3389/fpsyt.2022.983565)

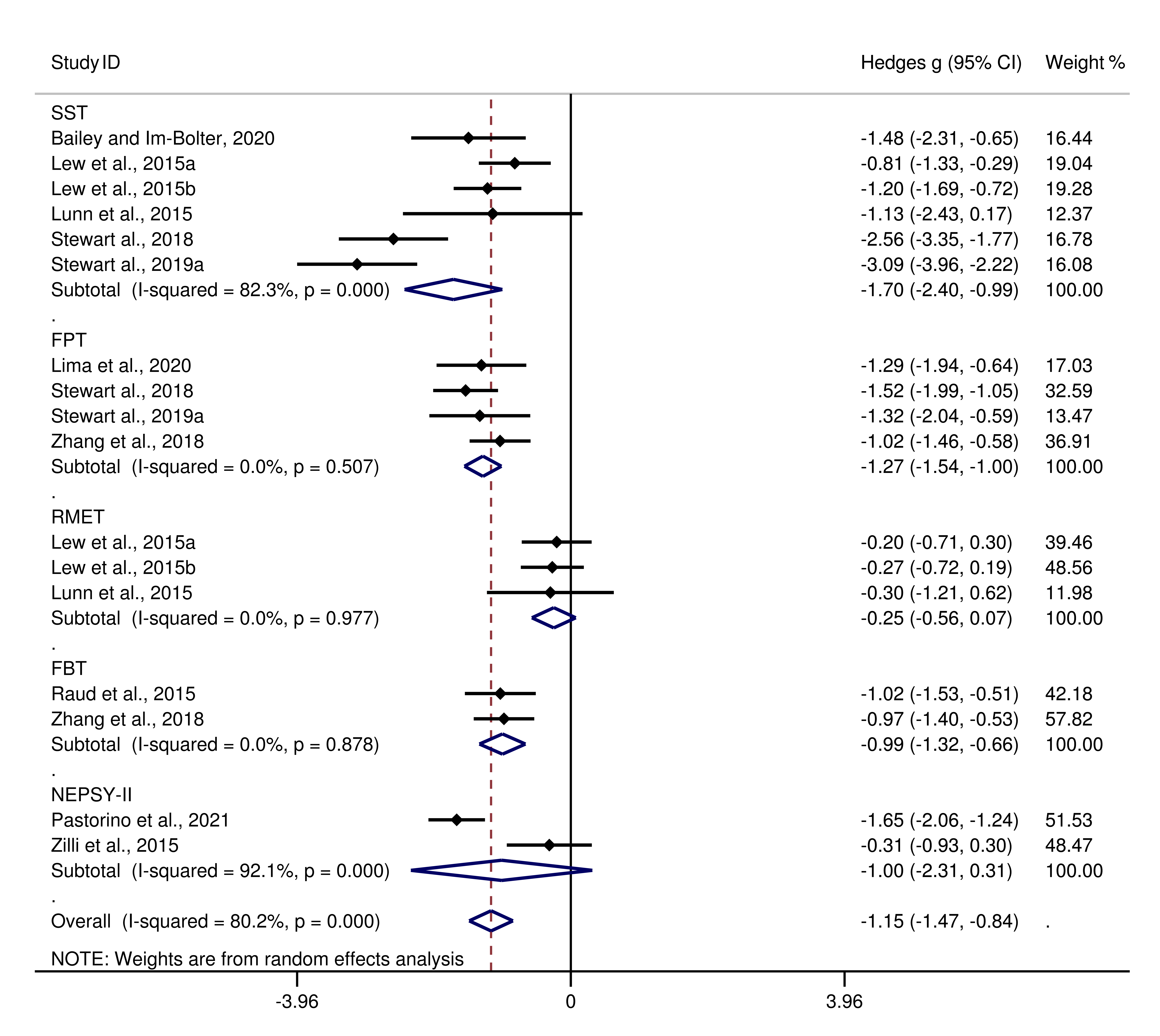

Supplement: Supplementary Figure 1 — Forest plots showing effect size estimates for individual ToM tasks differences between children and adolescents with epilepsy and healthy controls. [file Image_1.TIF]

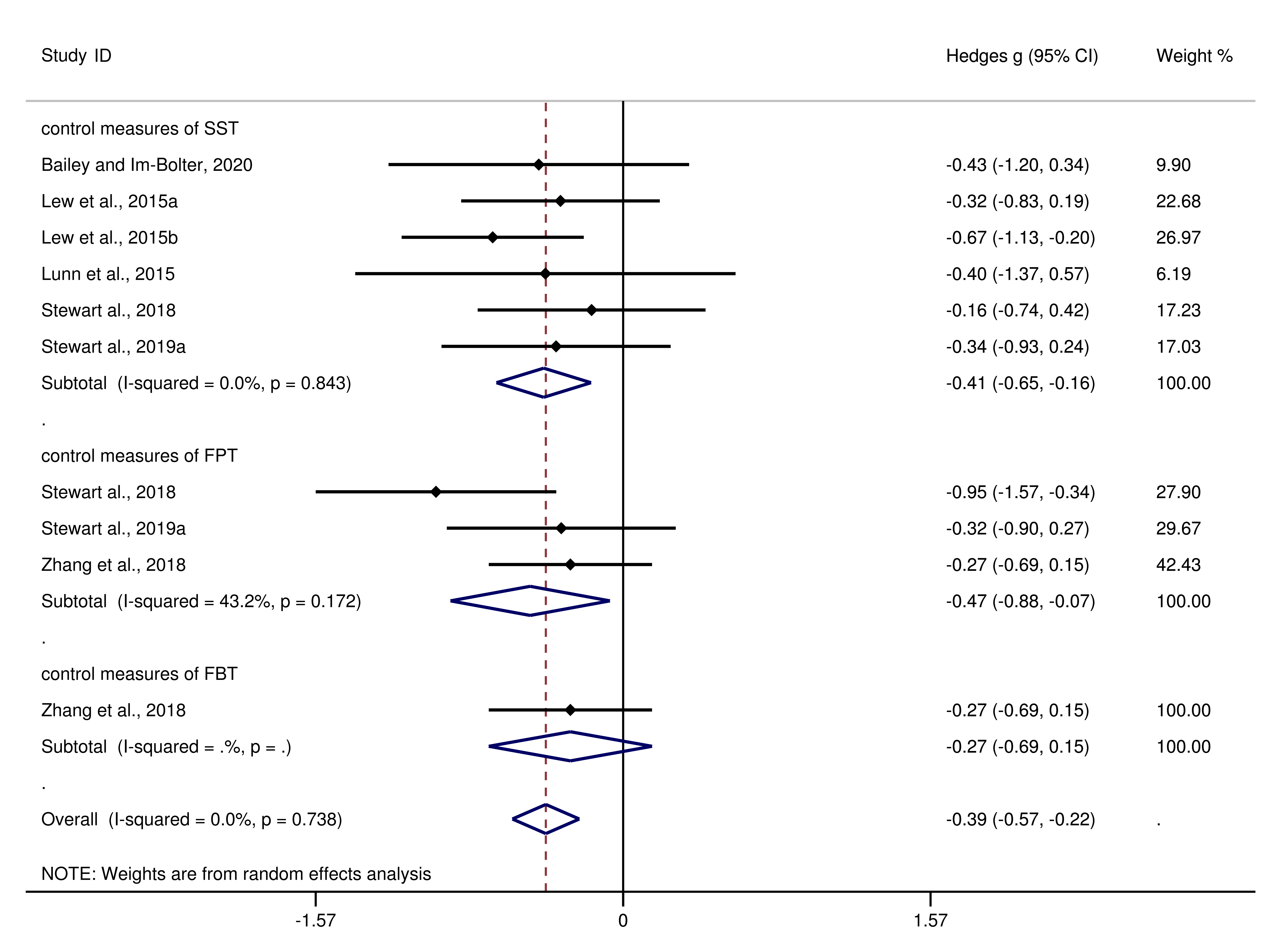

Supplement: Supplementary Figure 2 — Forest plots showing effect size estimates for control measures of ToM tasks differences between children and adolescents with epilepsy and healthy controls. [file Image_2.TIF]

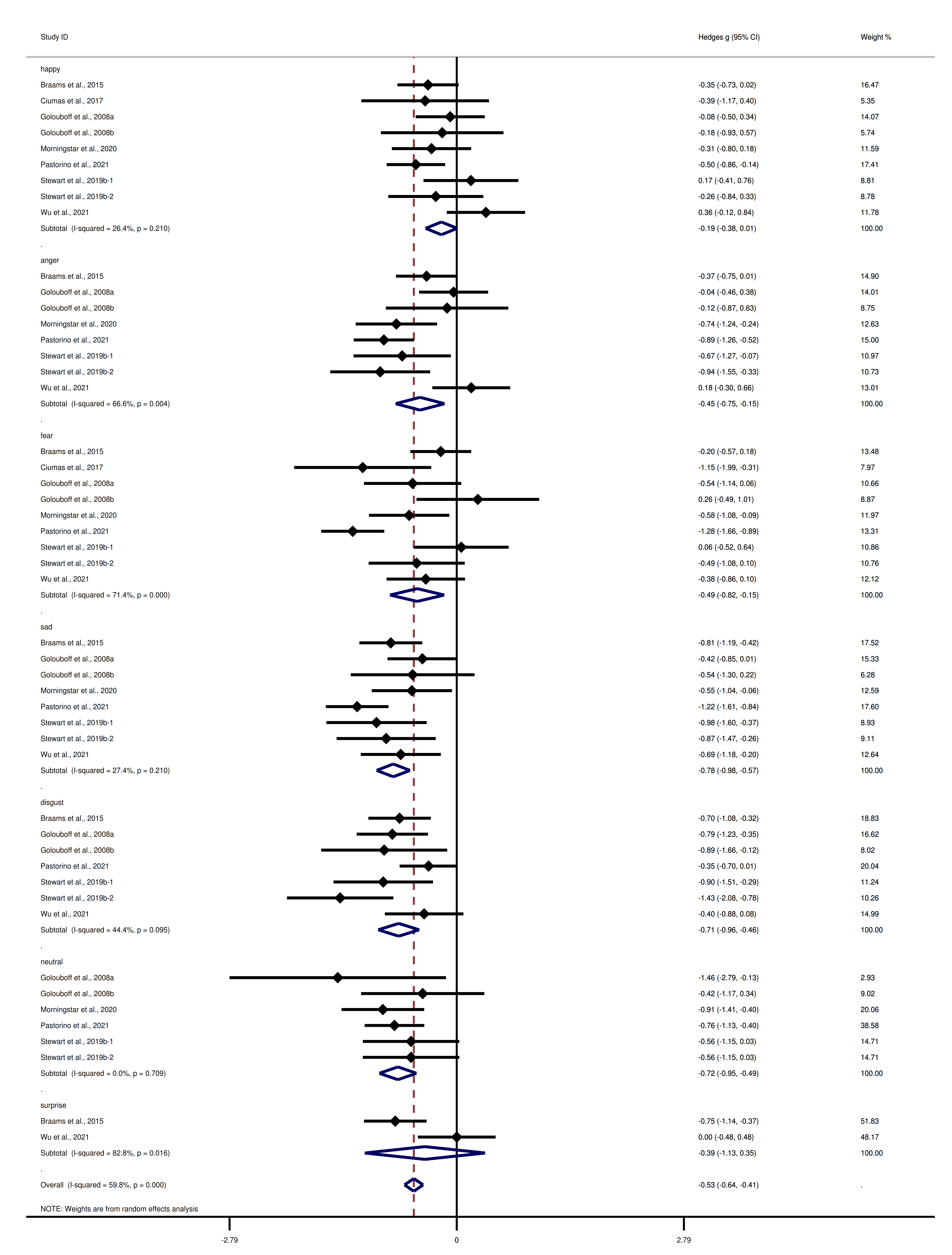

Supplement: Supplementary Figure 3 — Forest plots showing effect size estimates for individual emotions differences between children and adolescents with epilepsy and healthy controls. [file Image_3.TIF]
