# Supplementary material for: Social cognition in children and adolescents with epilepsy: A meta-analysis
Source: Front Psychiatry. 2022 Sep 15;13:983565. doi: 10.3389/fpsyt.2022.983565 (PMC9520261; doi:10.3389/fpsyt.2022.983565)
Supplement: Supplementary Table 1 — Mean effects for ToM and FER subcomponents comparing TLE and FLE against healthy controls and tests for publication bias. [file Table_1.docx]

**Supplementary Table 1.** **Mean effects for ToM and FER subcomponents comparing TLE and FLE against healthy controls and tests for publication bias**

| Test | *k* | *n* in TLE Group | *n* in HCs Group | *g* | 95% *CI* | |  | | Test for Heterogeneity | Assess risk of publication bias |
| --- | --- | --- | --- | --- | --- | --- | --- | --- | --- | --- |
|  |  |  |  |  | Lower | Upper | *z* value | *p* value | I^2^ Statistic,% | Egger’s test *p* value |
| overall ToM | 1 | 22 | 22 | -1.56 | -2.50 | -0.63 | -3.27 | 0.001 |  |  |
| cognitive ToM | 1 | 22 | 22 | -1.58 | -2.94 | -0.23 | -2.30 | 0.022 |  |  |
| affective ToM | 1 | 22 | 22 | -1.70 | -2.38 | -1.02 | -4.91 | < 0.001 |  |  |
| overall FER | 3 | 73 | 95 | -1.06 | -1.37 | -0.74 | -6.58 | < 0.001 | 0 | 0.914 |
|  |  | *n* in FLE Group | *n* in HCs Group |  |  |  |  |  |  |  |
| overall FER | 2 | 31 | 73 | -0.64 | -1.08 | -0.21 | -2.89 | 0.004 | 0 |  |

HCs = healthy controls; *CI* = confidence interval; FER = facial emotion recognition; TLE = temporal lobe epilepsy; FLE = frontal lobe epilepsy; *g* = Hedges g; ToM = theory of mind; *n* = the number; *k* = the number of studies.
